# Destruxin B Suppresses Drug-Resistant Colon Tumorigenesis and Stemness Is Associated with the Upregulation of miR-214 and Downregulation of mTOR/β-Catenin Pathway

**DOI:** 10.3390/cancers10100353

**Published:** 2018-09-25

**Authors:** Szu-Yuan Wu, Yan-Jiun Huang, Yew-Min Tzeng, Chi-Ying F. Huang, Michael Hsiao, Alexander T.H. Wu, Tse-Hung Huang

**Affiliations:** 1 Department of Radiation Oncology, Wan Fang Hospital, Taipei Medical University, Taipei 116, Taiwan; szuyuanwu5399@gmail.com; 2 Department of Internal Medicine, School of Medicine, College of Medicine, Taipei Medical University, Taipei 110, Taiwan; 3 Division of Colorectal Surgery, Department of Surgery, Taipei Medical University Hospital, Taipei Medical University, Taipei 110, Taiwan; colorectalman@gmail.com; 4 Department of Surgery, College of Medicine, Taipei Medical University, Taipei 110, Taiwan; 5 Department of Life Science, National Taitung University, Taitung 950, Taiwan; tzengym@gmail.com; 6 Institute of Biopharmaceutical Sciences, National Yang Ming University, Taipei 112, Taiwan; cyhuang1963@yahoo.com.tw; 7 Genomics Research Center, Academia Sinica, Taipei 11529, Taiwan; mhsiao@gate.sinica.edu.tw; 8 The PhD Program for Translational Medicine, College of Medical Science and Technology, Taipei Medical University, Taipei 115, Taiwan; 9 Graduate Institute of Medical Sciences, National Defence Medical Center, Taipei 114, Taiwan; 10 Department of Traditional Chinese Medicine, Chang Gung Memorial Hospital, Keelung 204, Taiwan; 11 School of Traditional Chinese Medicine, Chang Gung University, Taoyuan 204 Taiwan; 12 School of Nursing, National Taipei University of Nursing and Health Sciences, Taipei 23741, Taiwan

**Keywords:** colon cancer, destruxin B, cancer stem-like cells, 5-FU resistance, mTOR/β-catenin signaling and miR-214

## Abstract

Background: Drug resistance represents a major challenge for treating patients with colon cancer. Accumulating evidence suggests that Insulin-like growth factor (IGF)-associated signaling promotes colon tumorigenesis and cancer stemness. Therefore, the identification of agents, which can disrupt cancer stemness signaling, may provide improved therapeutic efficacy. Methods: Mimicking the tumor microenvironment, we treated colon cancer cells with exogenous IGF1. The increased stemness of IGF1-cultured cells was determined by ALDH1 activity, side-population, tumor sphere formation assays. Destruxin B (DB) was evaluated for its anti-tumorigenic and stemness properties using cellular viability, colony-formation tests. The mimic and inhibitor of miR-214 were used to treat colon cancer cells to show its functional association to DB treatment. In vivo mouse models were used to evaluate DB’s ability to suppress colon tumor-initiating ability and growth inhibitory function. Results: IGF1-cultured colon cancer cells showed a significant increase in 5-FU resistance and enhanced stemness properties, including an increased percentage of ALDH1+, side-population cells, tumor sphere generation in vitro, and increased tumor initiation in vivo. In support, using public databases showed that increased IGF1 expression was significantly associated with a poorer prognosis in patients with colon cancer. DB, a hexadepsipeptide mycotoxin, was able to suppress colon tumorigenic phenotypes, including colony and sphere formation. The sequential treatment of DB, followed by 5-FU, synergistically inhibited the viability of colon cancer cells. In vivo studies showed that DB suppressed the tumorigenesis by 5-FU resistant colon cells, and in a greater degree when combined with 5-FU. Mechanistically, DB treatment was associated with decreased the mammalian target of rapamycin (mTOR) and β-catenin expression and an increased miR-214 level. Conclusion: We provided evidence of DB as a potential therapeutic agent for overcoming 5-FU resistance induced by IGF1, and suppressing cancer stem-like properties in association with miR-214 regulation. Further investigation is warranted for its translation to clinical application.

## 1. Introduction

Colon cancer is one of the most prevalent malignancies in developed countries, as it is the third most common cancer causing death in the United States [1]. The underlying causes for the development of colon cancer are multifactorial, including risks from the environment, genetic background, and dietary habits [2]. Due to the complexity of the aetiology, the development of effective therapeutic agents has been limited, while cytotoxic chemotherapeutic agents, such as FOLFOX, remains as the standard treatment option. However, patients in advanced stages of the disease often develop resistance against treatment, so alternative approaches and/or targets are urgently needed. 

The existence of cancer stem-like cells (CSCs) has been identified in virtually all cancer types and is indicated to be involved in tumorigenic processes, including initiation, progression, metastasis, and drug resistance [3,4]. Growing evidence suggests that the microenvironment of the tumor plays a key role in generating and harboring CSCs—the exchange of cytokines and/or chemokines, tumor cells, and stromal cells promote a favorable microenvironment for tumorigenesis and the generation/maintenance of CSCs [5,6]. Insulin-like growth factor (IGF) signaling has been shown to be one of the key cytokines present in the colon tumor microenvironment, and is likely to be a promoter of colon tumorigenesis via promoting Wnt/β-catenin oncogenic signaling [7,8]. CSCs are characterized by their increased ability to resist treatments and to repopulate tumor mass post-interventions. Based on these premises, we intend to identify and screen for agents which can prevent the generation of and/or eliminate CSCs.

Chemicals derived from natural sources have been and still are the major source of new drug development, as exemplified by taxanes [9,10,11]. Destruxins are the most abundant secondary metabolites identified from the entomopathogenic fungus, *Metarhizium anisopliae*. Different members of destruxins have been identified and characterized. For instance, Destruxin A, B, and E (DA, DB, and DE) represents a class of cyclic depsipeptides, with the ability to disrupt macromolecular syntheses (such as DNA, RNA, and protein), and inhibit and prevent viral infections [12]. Our team has previously discovered that Destruxin B exhibited anti-cancer activities both in vitro and in vivo by suppressing Wnt/β-catenin signaling [13,14,15]. We further our previous findings by investigating DB’s potential as an inhibitor of colon CSCs, since Wnt/β-catenin signaling plays an instrumental role in maintaining the stemness in intestinal stem cells, and its increased activity has been shown to contribute colon tumorigenesis [16,17]. We first established that exogenous IGF1 treatment induced the exhibition of cancer stemness, including increased side-population (SP) and ALDH1 activity, accompanied by the increased resistance against the chemotherapeutic agent, 5-FU, and enhanced tumor-initiating ability in vivo. We found that DB treatment significantly suppressed IGF1-induced, CSC-associated properties, and acted synergistically with 5-FU to inhibit colon cancer proliferation. Mechanistically, DB-mediated anti-colon cancer activities was associated with the downregulation of stem/oncogenic markers, including β-catenin, c-Myc, and mTOR-associated molecules, while there was upregulation in a tumor suppressor, miR-214. In summary, we provide preclinical support and a rationale for the potential use of DB in conjunction with 5-FU in drug-resistant colon cancer patients in the future.

## 2. Materials and Methods

### 2.1. Cell Culture

The human colon cancer cell lines HCT116 and DLD-1 used in this study were purchased from the American Type Culture Collection (ATCC) (Manassas, VA, USA) and maintained and passaged according to the protocols provided. For CSC-enrichment experiments, IGF1 (200 ηg/mL, Millipore, Taipei, Taiwan) was added to the culture medium for 48 h (after the first 24 h, the culture medium was discarded and fresh medium containing IGF1 was replenished and cells cultured for an additional 24 h). IGF1-treated cells were then harvested for further analyses.

### 2.2. Stemness Assays 

To determine the enrichment of CSCs, the ALDEFLUOR™ assay (Stem Cell Technologies, Vancouver, BC, Canada) which measures the cellular aldehyde dehydrogenase (ALDH) activity was performed according to the manufacturer’s protocol. Accuri™ (BD BioSciences, Taibei, Taiwan) was used to determine and analyze ALDH1 activity of colon cancer cells used in this study. We also used a side-population assay as another methodology for examining the cancer stemness. In brief, the percentage of side-population cells (SP) were identified in both IGF1-treated HCT116 and DLD-1 cells, as well as the relative changes in SP percentage after DB treatments (1.0 and 2.0 μM) using a flow cytomerter, the FACSAria™ III sorter (BD Biosciences, Taipei, Taiwan). Verapamil (100 μM final concentration) was used to specifically inhibit ABC pumps 15 min prior to the addition of Hoechst. Verapamil was used as a control to confirm SP identification. 

### 2.3. In Vitro Cell Viability and Drug Test

Sulforhodamine B (SRB, Sigma-Aldrich, Taibei, Taiwan) assay was used for determining the cell viability of colon cancer cells under different conditions. Colon cancer cells were harvested and seeded into 96-well plates (5000 cells/well) for the assay. The cells were treated with different regimens: in the presence of 5-FU (ranging from 0 to 400 µM) and/or DB (ranging from 0 to 2 µM) for 48 h. In the case of the drug combination test, DB was added first for 24 h followed by the addition of 5-FU for an additional 24 h. Drug-treated cells were collected and fixed with 10% TCA. The fixed cells were then stained with 0.4% (*w*/*v*) SRB, which was then dissolved in 1% (*v*/*v*) acetic acid and solubilized in 20 mM Tris. The optical density (OD) of the samples was measured by a microplate reader (Molecular Devices, Sunnyvale, CA, USA) at 562 nm.

### 2.4. Western Blot Analysis

HCT116 and DLD-1 human colon cancer cells (parental and/or spheres) post different treatments were analyzed by SDS-PAGE and western blots using standard protocols. Protein samples (20–40 µg each well) were dissolved in sample buffer, denatured, and separated using 10% SDS-PAGE gels. The proteins were transferred onto nitrocellulose membranes and blocked in TBST (50 mM Tris-HCl pH 7.5, 150 mM NaCl, 0.2% Tween-20, 5% skim milk). Membranes were then incubated with respective primary and secondary antibodies. The protein–antibody interactions were determined by an enhanced chemiluminescence kit (ECL-Plus, Amersham Pharmacia Biotech, Piscataway, NJ, USA) and captured using the BioSpectrum^®^ Imaging System (Upland, CA, USA).

### 2.5. Colony-Forming and Tumor Sphere-Forming Assays

The colony-forming assay was carried out in the following conditions. Briefly, colon cancer cell lines, HCT116 and DLD-1 cells were seeded in six-well plates with (500 cells, 2.8 μM ovatodiolide, equivalent of IC10 values) and without ovatodiolide. The plates were then stained using 0.005% crystal violet, and the colonies were counted. The cells were allowed to grow for another week. The cells were then harvested, fixed, and counted. The migratory ability of the cells was examined using the Transwell migration assay (ThermoFisher, Taipei, Taiwan). To evaluate the self-renewal ability of cancer cells, we used a sphere-forming assay. Colon cancer cells were cultured under serum-deprived conditions and using Ultra-Low Attachment Plates (Corning Inc., Taipei, Taiwan). The culture conditions were modified slightly from Lo et al. [18]. Colon cancer cells (density: 10^4^ cells/mL) were cultured in a medium composed of 20 ng/mL epidermal growth factor (EGF), 10 ng/mL basic fibroblast growth factor (BFGF), 5 μg/mL insulin, and 0.4% Bovine Serum Albumin (BSA). After approximately 5–7 days of culture (depending on the cell type), tumor spheres were formed, and the numbers were counted under a phase-contrast microscope (40× magnification). The self-renewal ability was represented by the average number of spheres generated. The average sphere number formed was obtained from three different views. 

### 2.6. Animal Study

The in vivo experiments were performed by following the regulations of the Animal Care and User Committee at Taipei Medical University (Protocol #LAC-2017-0161). 8-week old NOD/SCID mice were purchased from BioLASCO (Taipei, Taiwan). Mice were housed in a specific pathogen-free (SPF) environment, and a week of acclimation was allowed prior to experiments. Two models were used in this study. The first model was to test the tumor-initiating ability of IGF-treated colon cancer cells in vivo. DLD-1 cells (50,000 cells per injection) cultured with and without exogenous IGF1 (100 ng/mL, 48 h) were subcutaneously injected. Tumor-initiating ability was measured and determined by the relative intensity of the bioluminescence (IVIS 200, Caliper, Taipei, Taiwan). In the second model, different drug regimens were tested. NOD/SCID mice were subcutaneously injected with 1 × 10^6^ DLD-1 colon cancer cells and randomly divided into 4 groups consisting of the vehicle control, DB (5 mg/kg, i.p injection, 5 times a week); 5-FU (25 mg/kg, i.p injection, 2 times a week), and the combination regimen: a decreased 5-FU concentration (10 mg/kg, i.p injection, 2 times a week) while maintaining DB dosage. Once the tumors became palpable, the starting tumor volume was recorded, and the treatment commenced. The tumor volume was recorded once a week with a standard formula: tumor size = (L × W^2^)/2, where L is the length and W is the width of the tumor. The body weight of the mice was monitored weekly. After the experiment, the animals were humanely sacrificed using cervical dislocation, and tumor samples were harvested for further analysis. All animal experiments were performed in accordance with the institutional guidelines for the care and use of laboratory animals approved by the Animal Care and User Committee at Taipei Medical University (Protocol #LAC-2017-0161) and the National Institute of Health guide for the care and use of laboratory animals.

### 2.7. Statistical Analysis

All experiments were performed at least in triplicates. Two-tailed t tests were used for analyses by GraphPad Prism software where a *p*-value < 0.05 was considered statistically significant.

## 3. Results 

### 3.1. Exogenous IGF1 Enriched Cancer Stem-Like Colon Cancer Cells and Induced 5-FU Resistance

**Figure 1:**
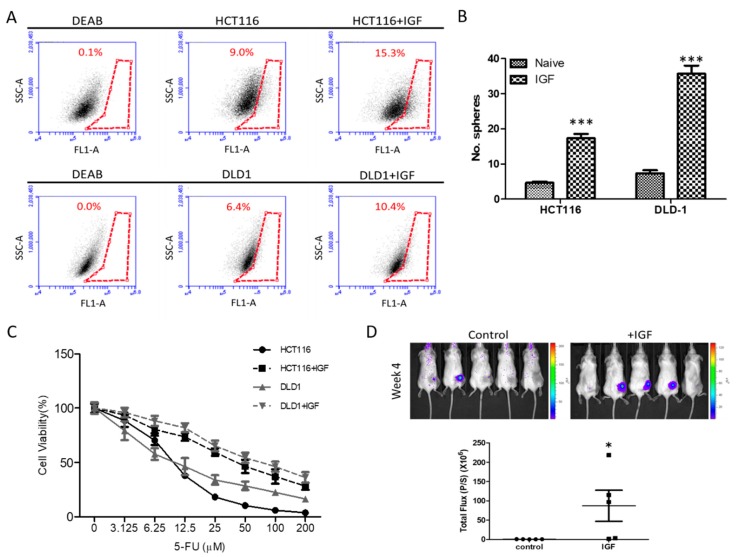
IGF1 promotes the generation of cancer stem-like (CSC) colon cancer cells. (**A**) Flow cytometry analysis showed that IGF1-treated colon cancer cells exhibited significantly higher ALDH1 activity. The DEAB Inhibitor of the ALDH enzyme was used as a control for ALDH1 analysis. (**B**) IGF1 treatment resulted in increased tumor sphere-generating ability in both DLD-1 and HCT116 cells, as compared to their naïve counterparts. (**C**) IGF1-treated HCT116 and DLD-1 cells became more resistant against 5-FU treatment. The insert describes the IC_50_ values obtained from both cell lines with and without IGF1 treatment. (**D**) Bioluminescence in vivo monitoring tumor-initiating ability of DLD-1 cells (50,000 cells per injection). Tumor spheres generated from IGF1-treated DLD-1 cells (100 ng/mL, 48 h) showed significantly enhanced tumor-initiating ability in vivo (60%) as compared to their naïve counterparts (20%), four weeks post inoculation. * *p ≤* 0.05; *** *p ≤* 0.001.

The insulin/IGF/mTOR system has been shown to play a key role in CRC development due to its complex involvement in the cancer’s cellular metabolism, proliferation, and differentiation [19,20]. Here, we showed that the exogenous IGF increased the cancer stem cell properties. Increased ALDH1 activity has been used to identify normal stem cells and/or cancer stem cells [21,22]. Here, we used flow cytometry to demonstrate increased cellular ALDH1 activity in IGF1-treated HCT116 and DLD-1 cells as compared to their naïve counterparts (Figure 1A). Notably, both HCT116 and DLD-1 cells pre-treated with IGF1 showed significantly increased ALDH1 activity. For instance, IGF1-treated HCT116 showed an approximately 6% increase in ALDH1 activity (Figure 1A). These IGF1-induced CRC cells were subsequently isolated and cultured under serum-deprived conditions. We found that these IGF1-treated CRC cells exhibited an enhanced ability to generate CSC-like spheres, as compared to their IGF1-naïve counterparts (Figure 1B). IGF1-treated CRC cells also demonstrated an increased ability to resist 5-FU treatment, as reflected by the higher IC_50_ values, than their naïve counterparts (Figure 1C). For instance, the IC50 values of 5-FU in naïve HCT11 increased from 11.2 to 41.9 µM, while it was from 15.9 to 60.0 µM in DLD-1 cells (Figure 1C). More importantly, we demonstrated that IGF1 treatment promoted tumor initiation in vivo. DLD-1 tumor spheres cultured with and without IGF1 were injected into NOD/SCID mice for evaluation. We found that IGF1-cultured tumor spheres appeared to initiate tumorigenesis with a significantly higher rate than their counterparts without IGF1 treatment (60% versus 20%), respectively (Figure 1D). 

### 3.2. IGF1 and β-Catenin Expression Is Associated with Drug Resistance and Poor Prognosis in Colon Cancer Patients

**Figure 2:**
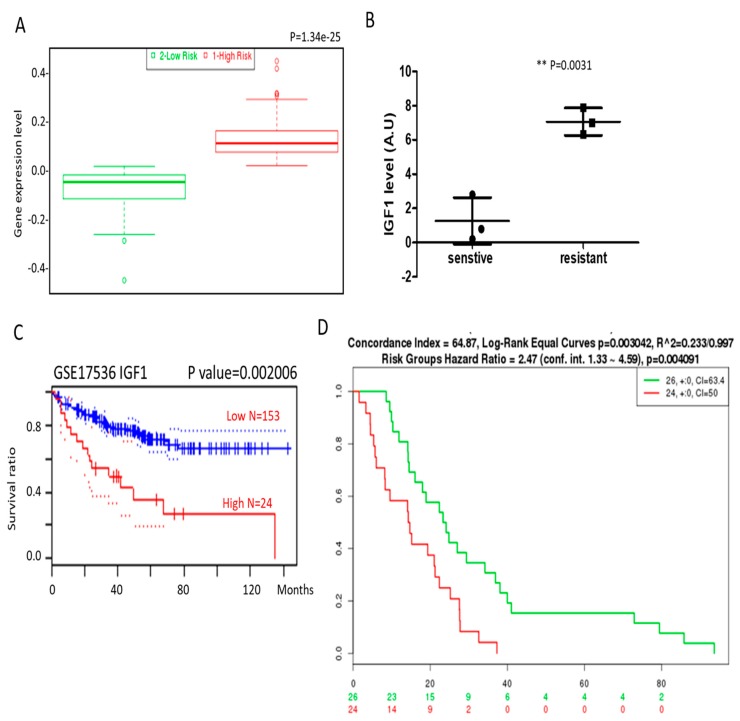
Increased IGF1 and β-catenin expression is associated with a higher incidence of colon cancer and poorer prognosis. (**A**) Using the SurvExpress database, we found that an increased IGF1 mRNA level was associated with a higher risk of developing colon cancer. (**B**) Our GEO (Gene Expression Omnibus) database analysis showed that a significantly higher level of IGF1 mRNA was detected in methotrexate-resistant colon cancer cells than the more sensitive counterparts. (**C**) The Kaplan-Meier survival curve obtained from a small cohort (N = 177) showed that increased IGF1 expression is significantly associated with a lower survival ratio in patients with colon cancer. (**D**) Disease-free survival (DFS) analysis of the GSE14333 cohort indicates a shorter DFS in patients with colon cancer expressing a higher level of both IGF1 andβ-catenin (CTNNB1). (** *p* ≤ 0.01 as statistically significant).

To add support to our in vitro and in vivo results, we analyzed the public databases and demonstrated that the IGF1 mRNA level was significantly higher in patients with colon cancer [23] (Figure 2A). In addition, an increased IGF1 mRNA was detected in the methotrexate-resistant colon cancer cells [24] (Figure 2B). Analysis from a cohort of colon cancer patients (GSE17536 series) [25] using PrognoScan software showed that higher IGF1 expression in the patients was significantly associated with shorter survival (Figure 2C). Using another database (GSE14333) [26], we found that increased expression in both IGF1 and CTNNB1 (β-catenin) in patients was associated with significantly shorter disease-free survival (Figure 2D). 

### 3.3. DB Treatment Suppressed Drug-Resistant Colon Tumorigenesis and Stemness

**Figure 3:**
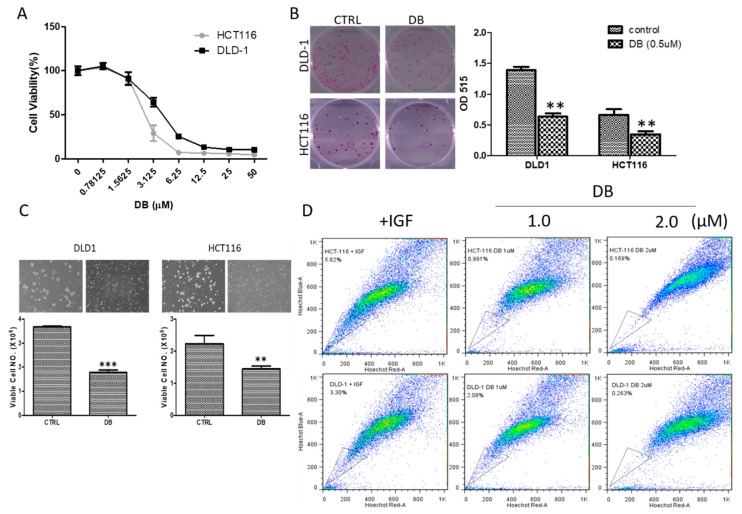
Destruxin B (DB) treatment suppressed tumorigenesis of 5-FU-resistant colon cancer cells. (**A**) Cell viability assay of IGF1-trained colon cancer cell lines in response to DB treatment. Both 5-FU resistant HCT116 and DLD-1 cells were sensitive towards DB treatment, as reflected by their respective IC50 values, 3.04 and 4.99 µM. Colony (**B**) and tumor sphere (**C**) formations were also significantly reduced by the DB treatment in both cell lines. (**D**) Side-population (SP) assay demonstrated that DB dose-dependently decreased the percentage of SP cells in both IGF1-cultured HCT-116 and DLD-1 cells. (** *p* ≤ 0.01 and *** *p* ≤ 0.001).

Our previous studies have demonstrated that DB treatment significantly suppressed tumorigenesis in different cancer types [13,14,15,27,28]. In this study, we examined the potential inhibitory effect of DB using 5-FU resistant colon cancer cells, induced by IGF1. We found that DB treatment significantly suppressed the viability of both IGF-treated HCT116 and DLD-1 (Figure 3A). For instance, DB treatment achieved the half maximal inhibitory effect on the cell viability on HCT116 and DLD-1 cells at 3.04 and 4.99 µM, respectively. In addition, DB (0.5 µM) markedly inhibited the formation of colonies and tumor spheres in both IGF-treated colon cancer cell lines (Figure 3B,C). The percentage of the side-population activity in both IGF1-treated HCT116 and DLD-1 cells was also dose-dependently reduced (Figure 3D). For example, IGF1-cultured HCT-116 cells were originally found to contain approximately 5.8% of SP cells, but with DB treatment (at 2 µM, 24 h), the percentage of SP cells significantly reduced down to approximately 0.17% (upper panels, Figure 3D).

### 3.4. DB and 5-FU Synergistically Suppresses Viability of Colon Cancer Cells

**Figure 4:**
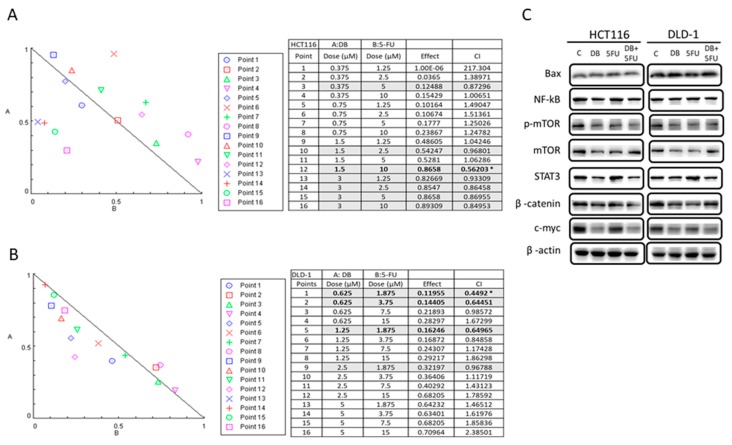
The combination of DB and 5-FU suppressed colon cancer viability. HCT116 (**A**) and DLD-1 (**B**) colon cancer cells were subjected to different concentrations of DB and 5-FU treatment. The combination index (CI) defines synergism (CI < 1), the additive effect (CI = 1), and antagonism (CI > 1). The asterisk depicts the most effective combination of DB and 5-FU. (**C**) Western blot analysis showed that the DB and 5-FU combination led to reduced expression of oncogenic markers (mTOR, STAT3), stemness markers (β-catenin, c-Myc), and an increased expression of the pro-apoptotic marker, Bax.

Next, we examined the plausibility of combining DB and the clinical chemotherapeutic agent 5-FU for treating colon cancer cells. We tested different combinations of DB (ranging from 0–5 µM) and 5-FU (from 0–15 µM) to determine the combination index (CI). Using CompuSyn software, we plotted isobolograms derived from the different concentrations of DB versus 5-FU. Several specific combinations of DB and 5-FU synergistically inhibited the cell viability of both HCT116 and DLD-1 (Figure 4A,B respectively). In support, our Western blot analysis indicated that DB treatment led to the decrease in IGF downstream markers, including STAT3, mTOR, β-catenin, NF-kB, and c-Myc expression, while there was an increase in Bax expression (Figure 4C). Numbers in bold and marked by an * indicates the most effective combination.

### 3.5. In Vivo Demonstration of DB Treatment Enhanced 5-FU Efficacy

**Figure 5:**
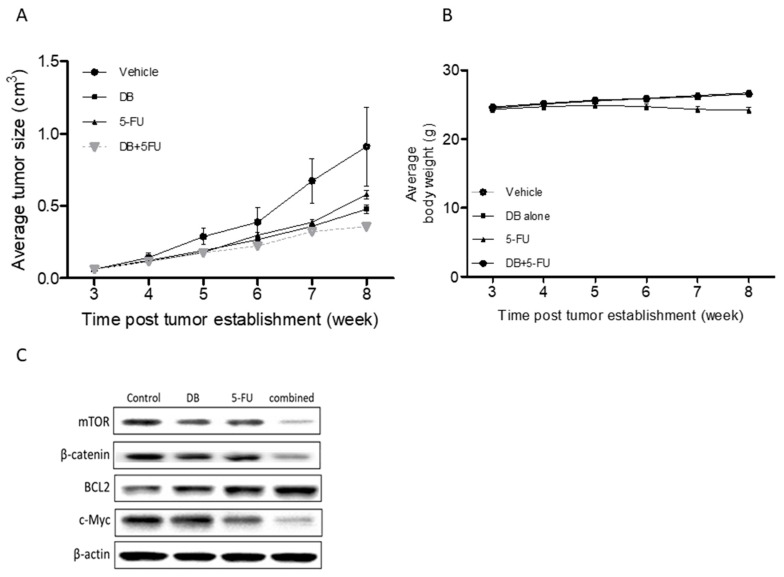
The DB and 5-FU combination suppressed 5-FU resistant DLD-1 tumorigenesis in vivo. (**A**) The tumor-size curve over time demonstrates that DB alone was more effective than delaying IGF1-treated DLD-1 cells, while the combination of DB and 5-FU was the most effective. (**B**) The body-weight curve over time shows that the average body weight of all the animals were not significantly different. (**C**) Western blot analysis of tumor samples harvested. Oncogenic markers, including mTOR, β-catenin, and c-Myc were all suppressed, while Bcl2 increased under DB treatment and showed up more prominently in the combination treatment group.

Subsequently, we aimed to validate our in vitro data using the xenograft mouse colon-cancer model. IGF1-treated DLD-1 cancer cells were subcutaneously transplanted into NOD/SCID mice, and the tumor-bearing mice were then divided into four groups: sham control, DB (5 mg/kg, 5 times/week), 5-FU (25 mg/kg, 2 times/week), and the combination of DB and 5-FU. Mice which received DB or 5-FU alone appeared to exhibit a similar tumor burden, whereas the tumor burden appeared to be lowest in the combination group (Figure 5A). We also monitored the body weight of the test subjects and did not find any significant difference among them (Figure 5B), suggesting no apparent systematic toxicity in all treatment regimens. A western blot analysis was performed on the tumor samples harvested. We found that DB-treated samples exhibited a markedly reduced expression of mTOR, c-Myc, and β-catenin, while there was an increase in Bcl2 (Figure 5C). A similar observation was made in the 5-FU group, but not as significant as that of the DB-treated group. Treatment using a combination of DB and 5-FU showed the lowest expression in the aforementioned oncogenic markers.

### 3.6. An Increased MicroRNA-214 Level Was Associated with DB Treatment

**Figure 6:**
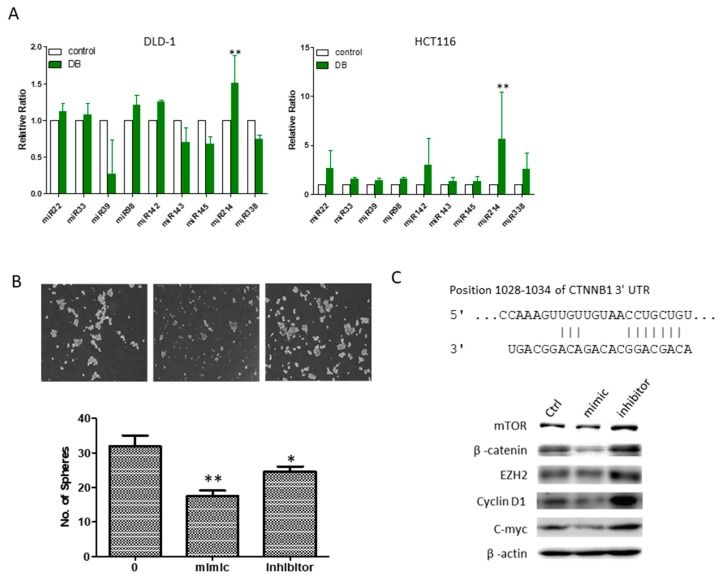
DB treatment was associated with an elevated miR-214. (**A**) Profiling of microRNA expression pattern in DB-treated colon cancer cells. DB treatment led to a significantly increased miR-214 level in both HCT116 and DLD-1 cells. (**B**) Increased miR-214 expression by miR-214 mimic was associated with the suppression of the generation of HCT-116 tumor spheres, while the miR-214 inhibitor reversed the suppressive effect. (**C**) The potential 3′UTR site of CTNNB1 for miR-214 binding was detected using TargetScan software. Western blot analysis showed that miR-214 mimic treatment led to a decreased expression of mTOR, β-catenin, EZH2, cyclin D1, and c-Myc, whereas an increased expression of these markers was found in HCT-116 cells treated with the miR-214 inhibitor. (* *p* ≤ 0.05; ** *p* ≤ 0.01).

Finally, we examined a small panel of microRNAs (miRs) as an attempt to explore the potential underlying molecular mechanism associated with DB treatment. Among the different miRs examined, the miR-214 level significantly increased post-DB treatment in both DLD-1 and HCT-116 cell lines (Figure 6A). Phenotypically, increasing the level of miR-214 was associated with the decreased ability of HCT-116 tumor sphere formation (Figure 6B), while the miR-214 inhibitor reversed the miR-214-associated anti-tumor sphere-forming ability (Figure 6B). We identified a potential binding site of miR-214 in the 3′UTR of CTNNB1 (β-catenin, Figure 6C). More importantly, DLD-1 cells treated with miR-214 mimic molecules showed a concomitant decreased expression of several oncogenic and stemness markers, including β-catenin, mTOR, EZH2, cyclin D1, and c-Myc, while the reverse was true when cells were treated with the miR-214 inhibitor (Figure 6D). 

## 4. Discussion 

The generation of cancer stem-like cells (CSCs) has shown to occur both experimentally and clinically [29]. It is believed that treatment failure and disease progression have been closely associated with the presence of CSCs. However, how CSCs are generated still remains unclear and the development of an antagonist proves difficult. Accumulating evidence has pointed out that de-regulation of the insulin growth factor (IGF)-signaling cascade not only plays an instrumental role in cellular growth and metabolism, but also colon tumorigenesis [30,31]. Based on this unmet medical need, we first examined the role of IGF signaling in its relationship with CSC generation. We established 5-FU-resistant colon cancer cell lines by culturing these cells with exogenous IGF1. The exogenous IGF1 promoted CSC phenotypic changes in colon cancer cells. More importantly, we demonstrated that IGF1-cultured DLD-1 cells exhibited an enhanced tumor-initiating ability in vivo as compared to their naïve counterparts. Our analyses using the public database also indicated that an increased IGF1 mRNA was identified in patients with colon cancer as well as a shorter survival time, as compared to patients with a lower IGF1 mRNA level; HT29 colon cancer cells resistant to methotrexate expressed a significantly higher level of IGF1 mRNA as compared to their sensitive counterparts. A recent study corroborates with our view that IGF-signaling promotes the epithelial-to-mesenchymal transition (EMT) and stemness in colon cancer [32]. 

Fluorouracil (5-FU) is a standard chemotherapeutic drug for treating different types of cancer, including colon cancer. Unfortunately, tumor cells often develop resistance against it, resulting in relatively low efficacy (approximately 40%). Our observations where IGF1 promoted the phenotypic manifestation of colon CSCs, namely resistance against 5-FU, presents an important consideration when developing novel therapeutics. 

Statistically, in the area of cancer research, over the period of 1940 to 2014, of the 175 small molecules approved, 131 (75%) were either natural products or directly derived therefrom [33]. Thus, natural resources still remain as a valuable pool for anti-cancer drug discovery. Destruxin B (DB) is a cyclic depsipeptide produced by various species of fungi, and has been previously shown to inhibit proliferation and induce apoptosis in different cancer cells [12,13,14], making it a drug candidate for further development. Here, we showed that DB treatment not only suppressed colon tumorigenesis, but also CSC phenotypes. DB-medicated CSC-inhibitory effects were associated with the decreased expression of the IGF downstream oncogenic marker, mTOR/STAT3, as well as the stemness marker, β-catenin, both of which have been reported to contribute to treatment failure and the recurrence of colon cancer [23,34,35]. 

More importantly, we provided two lines of in vivo support for treating colon cancer with DB, either alone or in combination with 5-FU. DB alone could significantly delay DLD-1 tumorigenesis in the xenograft mouse model; when combined with 5-FU, the anti-cancer effect was even more pronounced, corroborating with the in vitro data where the combination of 5-FU and DB (at a lower dosage) could synergistically suppress the viability of colon cancer cells. Clinically, reduced dosage of 5-FU could prevent the development of side-effects and improve patient compliance. 

In another experiment, we showed that a single DB pre-treatment (below IC50) could significantly reduce the tumor-initiating ability of IGF1-cultured DLD-1 colon CSC-like cells in vivo. Mechanistically, DB treatment was associated with the decreased expression of a key CSC marker, β-catenin, which has been shown to be aberrantly increased in malignant colon cancer cells and associated with the generation of drug-resistance [36,37]. DB-mediated suppression of β-catenin was also accompanied with an increased miR-214 level, which has a potential binding site at β-catenin’s 3′UTR. These findings implied that DB pre-treatment led to the reduced stemness of the CSCs, thereby reducing its tumor-initiating ability via suppressing the CSC-associated marker, β-catenin. On this point, DB may be used to prevent the development of colon cancer in high-risk individuals or for prolonging progression-free time for patients with colon cancer. 

## 5. Conclusions

We provided evidence for the functional roles of DB as not only a therapeutic candidate drug for colon cancer, but also a CSC inhibitor. DB-mediated functions were attributed to its ability to suppress several major oncogenic pathways, namely mTOR/Akt, c-Myc, NF-kB, and the stemness maker, β-catenin. Further investigation is warranted in order for DB to be repurposed for treating drug-resistant colon cancer. 

## Abbreviations

DB	Destruxin B
CSCs	Cancer stem-like cells
5-FU	Fluorouracil
SP	Side population
IGF1	Insulin-likegrowth factor 1
mTOR	The mammalian target of rapamycin
